# Neuroprotection by upregulation of the major histocompatibility complex class I (MHC I) in SOD1^G93A^ mice

**DOI:** 10.3389/fncel.2023.1211486

**Published:** 2023-08-30

**Authors:** Ana Laura M. R. Tomiyama, Luciana Politti Cartarozzi, Lilian de Oliveira Coser, Gabriela Bortolança Chiarotto, Alexandre L. R. Oliveira

**Affiliations:** Department of Structural and Functional Biology, Institute of Biology—University of Campinas (UNICAMP), Campinas, Brazil

**Keywords:** amyotrophic lateral sclerosis, IFN β, ALS therapy, MHC-I, gliosis, neuroprotection

## Abstract

Amyotrophic lateral sclerosis (ALS) is a neurodegenerative disease that progressively affects motoneurons, causing muscle atrophy and evolving to death. Astrocytes inhibit the expression of MHC-I by neurons, contributing to a degenerative outcome. The present study verified the influence of interferon β (IFN β) treatment, a proinflammatory cytokine that upregulates MHC-I expression, in SOD1^G93A^ transgenic mice. For that, 17 days old presymptomatic female mice were subjected to subcutaneous application of IFN β (250, 1,000, and 10,000 IU) every other day for 20 days. Rotarod motor test, clinical score, and body weight assessment were conducted every third day throughout the treatment period. No significant intergroup variations were observed in such parameters during the pre-symptomatic phase. All mice were then euthanized, and the spinal cords collected for comparative analysis of motoneuron survival, reactive gliosis, synapse coverage, microglia morphology classification, cytokine analysis by flow cytometry, and RT–qPCR quantification of gene transcripts. Additionally, mice underwent Rotarod motor assessment, weight monitoring, and neurological scoring. The results show that IFN β treatment led to an increase in the expression of MHC-I, which, even at the lowest dose (250 IU), resulted in a significant increase in neuronal survival in the ALS presymptomatic period which lasted until the onset of the disease. The treatment also influenced synaptic preservation by decreasing excitatory inputs and upregulating the expression of AMPA receptors by astrocytes. Microglial reactivity quantified by the integrated density of pixels did not decrease with treatment but showed a less activated morphology, coupled with polarization to an M1 profile. Disease progression upregulated gene transcripts for pro- and anti-inflammatory cytokines, and IFN β treatment significantly decreased mRNA expression for IL4. Overall, the present results demonstrate that a low dosage of IFN β shows therapeutic potential by increasing MHC-I expression, resulting in neuroprotection and immunomodulation.

## 1. Introduction

Amyotrophic lateral sclerosis (ALS) is a neurodegenerative disease that affects motoneurons, causing progressive muscle atrophy and paralysis. From the onset of the symptoms, clinical signs such as muscle weakness in the upper and lower limbs, slurred speech, and dysphagia present a progressive worsening until the patient’s death, usually within the first 5 years of diagnosis, which usually occurs due to respiratory muscle paralysis, especially the diaphragm (Malessa et al., 1991; Brooks, 1994; Rowland and Shneider, 2001; Miller et al., 2012).

Amyotrophic lateral sclerosis comprises approximately 90% of motoneuron disease (MND)-diagnosed cases. The worldwide incidence rate is, on average, 1.75 cases per 100,000. The more common mutations associated with ALS are related to C9orf72, SOD1, and TARDBP genes, characterizing familial ALS. However, in a significant percentage of cases, the disease occurs spontaneously, characterizing sporadic ALS, which etiology remains unknown. Early diagnosis is uncertain since symptoms only become evident when approximately 40% of motoneurons have already degenerated, which significantly reduces the chance of successful treatment (Brooks, 1994; Marin et al., 2017).

Patients, regardless of disease etiology and mutation, suffer from neuronal excitotoxicity, which contributes to neuronal damage and motoneuron death. The mutations result in a series of chemical and cellular reactions that are not yet completely known and lead mainly to glutamatergic excitotoxicity in the central nervous system (CNS) (Hardiman et al., 2017; van Es et al., 2017). Degeneration of motoneurons is accentuated by changes in the neuropile, affecting glial cells, such as in astrocytes, which may assume a toxic profile, favoring disease progress. The control of excitotoxicity is a pivotal role of astrocytes, as they are responsible for providing trophic support to motoneurons, in addition to mediating the rapid reuptake of glutamate after its release from the presynaptic inputs (Zhao et al., 2004; Boillée et al., 2006). An interplay among glial cells also takes place, since astrocytes expressing mutations characteristic of ALS increase microglial activation, upregulating the inducible nitric oxide synthase (iNOS), leading to further damage to motoneurons and accelerating disease progression through a non-cellular autonomous mechanism (Nagai et al., 2007; Yamanaka et al., 2008).

Recent literature has shown that astrocytes from individuals with ALS lead to decreased expression of the major histocompatibility complex of class I (MHC-I) in motoneurons. In contrast, upregulating MHC-I levels protects motoneurons from the toxic effects of astrocytes, suggesting that the neuronal expression of MHC-I modulates the susceptibility to toxicity caused by astrocytes and may delay disease progression (Nardo et al., 2016; Song et al., 2016). This is in line with the fact that MHC-I upregulation after motoneuron axotomy activates a series of protective mechanisms and facilitates axonal regeneration (Chiarotto et al., 2017).

Major histocompatibility complex of class I is a transmembrane heterodimer cell surface receptor, composed of an α chain, which is folded into three extracellular globular domains (α1, α2, and α3) coupled to the obligatory β2 microglobulin (β2 m) subunit (Maenaka and Jones, 1999). In addition to the well-established role in the immune system, it has become clear that MHC-I plays a non-immune role in CNS development (Elmer and McAllister, 2012), and is involved in mechanisms of synaptic plasticity and axonal regeneration (Corriveau et al., 1998; Oliveira et al., 2004; Castelli-Haley et al., 2010; Zanon et al., 2010; Inácio et al., 2012; Goodin, 2013; Cebrian et al., 2014; Lee et al., 2014; Mahurkar et al., 2014; Bombeiro et al., 2016, 2017; Inacio et al., 2016; Cartarozzi et al., 2019), acting as a neuron-neuron and neuron-glia communication mechanism, modulating the cellular response to injury and disease (Oliveira et al., 2004; Zanon and Oliveira, 2006).

Importantly, MHC-I expression in motoneurons of patients with both types of ALS is almost absent as compared to healthy individuals, suggesting that astrocytes, in addition to exerting toxic and neurodegenerative effects, also induce downregulation of MHC-I, preventing any neuroprotective function (Song et al., 2016).

It has already been shown that MHC-I expression can be induced by treatment with the proinflammatory cytokine interferon β-1b (IFN β) and that animals submitted to peripheral nerve injury that received treatment with IFN β presented faster motor recovery (Zanon and Oliveira, 2006; Zanon et al., 2010). Interferons are cytokines that act on the immune system by increasing the expression of MHC-I in most tissues, enhancing the detection of infected cells, and thus inhibiting viral replication (Israel et al., 1989; Sen, 2001).

Influence of interferon β is used in clinical practice for multiple sclerosis immunomodulatory treatment; although the mechanism of action is not completely clear, it is well accepted that IFN β may interfere in T lymphocyte activation and proliferation, enhancing apoptosis of autoreactive T cells, thus modulating anti-inflammatory cytokines, as well as the permeability of the blood-brain barrier (Lam et al., 2008). Although previous work evaluated the efficacy of recombinant interferon β-1a in ALS patients, without significantly positive results, the high dosage used (10 mIU) may have produced many side effects that obscured any potential benefit (Beghi et al., 2000).

In this sense, IFN β low-dose treatment has not been tested in SOD1^G93A^ mice so the upregulation of MHC I could influence the glial reaction and motoneuron survival (Dhib-Jalbut and Marks, 2010). Therefore, the present study aimed at studying the effects of increased MHC-I expression in SOD1^G93A^ mice through treatment with low doses of IFN β and analyzed whether such upregulation leads to a neuroprotective effect, acting on the modulation of the motoneuron environment, and reducing the toxicity of ALS reactive glia.

## 2. Materials and methods

### 2.1. Mice

For this study, forty female SOD1^G93A^ (B6SJL) and 10 non-transgenic (NTG) littermates were used. SOD1^G93A^ mice overexpress the human gene SOD1 with Gly93 → Ala mutation (Gurney, 1994). All mice were kept at the Laboratory of Nerve Regeneration, Institute of Biology, University of Campinas. Mice were maintained in appropriate microisolators under a 12 h light-dark cycle with controlled temperature and humidity and water and food were provided *ad libitum*.

The colony was maintained by crossing transgenic males with wild-type females. The litter was genotyped by PCR, as recommended by the Jackson Laboratory manual.

DNA extraction and amplification were performed using the Extract-N-Amp™ Tissue PCR Kit (Sigma Aldrich) according to the manufacturer’s instructions. The primers used are described in Table 1.

**TABLE 1:** Primer sequences for mouse genotyping.

Primers	Sequence
Human SOD forward	5′ CAT CAG CCC TAA TCC ATC TGA 3′
Human SOD reverse	3′ TCT TAG AAA CCG CGA CTA ACA ATC 5′
Mouse SOD forward	5′ GCA ATC CCA ATC ACT CCA CAG 3′
Mouse SOD reverse	3′ GTC CAT GAG AAA CAA GAT GAC 5′

After genotyping, the mice were distributed into five experimental groups. The non-transgenic mice (NTG) were used as a control group, and transgenic mice were divided into a vehicle and an IFN β treatment group (Table 2).

**TABLE 2:** Experimental groups and distribution of animal numbers for each technique.

	Groups	No of animals per technique	
**Pre-symptomatic period (90 days of life)**		**Immunofluorescence motoneuron survival**	**qRT–PCR, flow cytometry, and microglial morphology**
	NTG (non-transgenic)	5	5
	Vehicle	5	5
	IFNβ 250 IU/day	5	5
	IFNβ 1,000 IU/day	5	*
	IFNβ 10,000 IU/day	5	*
Initial symptomatic period (100 days of life)	NTG (non-transgenic)	5	
	Vehicle	5	
	IFNβ 250 IU/day	5	

*Corresponds to the experimental group with the most effective dose.

All experiments and animal handling were approved by the Institutional Committee for Ethics in Animal Experimentation (CEUA, Institute of Biology, UNICAMP, protocol no 5323–1/2019 and n° 5878–1/2021).

### 2.2. Drug and treatment

Influence of interferon β treatment was initiated on the 70th day of life (presymptomatic period), every other day by subcutaneous injection, until the 90th day, with three different IFN β doses: 250, 1,000, and 10,000 IU, and until 100 days of life, only with the most efficient IFN β dose. Mice belonging to the vehicle group received a saline injection.

### 2.3. Animal survival assessment

Disease progression was evaluated in all transgenic mice every 3 days between the 70th and 90th day of life, always between 13:00 and 16:00 to avoid diurnal variations (Gurney, 1994).

The parameters measured included body weight, neurological score, and latency to fall in the Rotarod test.

#### 2.3.1. Disease evolution

Bodyweight and neurological scores were monitored to detect signs of muscle atrophy and disease progression. The neurological score of 0 to 4 was evaluated as described in the strain manual of Jackson Laboratory: Working with ALS Mice Guidelines for preclinical testing and colony management the Jackson Laboratory.^1^

Stage 0: the animal performs the full extension of the hind limbs to the body when it is suspended by the tail.

Stage 1: partial collapse of the extension of the hind limbs toward the body (weakness) or tremor of the hind limbs during suspension by the tail.

Stage 2: finger curl or any part of the paw is dragging at least twice while walking 12 inches.

Stage 3: rigid paralysis or minimal joint movement, not using the paws for generation of the forward movement.

Stage 4: the animal is unable to straighten within 30 s of being positioned on both sides.

#### 2.3.2. Motor performance

For Rotarod (EFF 412, Insight, Brazil) tests, mice had up to 8 min to remain in the rotating bar at a constant speed of 5 rpm. The time until the mice dropped from the cylinder was recorded.

### 2.4. Tissue preparation for histological examination

After de end of the treatments, mice were anesthetized with Anasedan (xylazine, Ceva, Brazil, 10 mg/kg) and Dopalen (ketamine, Ceva, Brazil, 50 mg/kg) and subjected to transcardial perfusion with 0.9% saline in 0.1 M phosphate buffer (PBS), followed by fixative solution (4% paraformaldehyde in 0.1 M phosphate buffer—PB; pH 7,4). The lumbar spinal cords were removed, post-fixed in the same fixative solution for 12 h at 4 °C, washed with phosphate buffer (PB), and sequentially cryopreserved in 10, 20, and 30% PB-sucrose solution (12 h in each concentration).

Samples were individually frozen in n-hexane cooled in liquid nitrogen at −35°C. Transverse sections (12 μm thick) of the lumbar spinal cords were obtained in a cryostat (Microm HM525), transferred to gelatin-coated slides, dried at room temperature for 30 min, and stored at −20°C until use.

**FIGURE 1:**
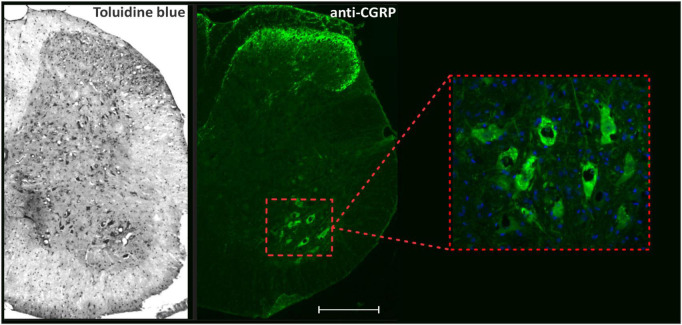
Panoramic view of transverse sections of the spinal cord correlating toluidine blue staining with anti-CGRP-positive motoneurons. The inset shows the motoneuron pool, the region where neuronal survival and immunofluorescence analyzes were performed. Scale bar = 150 μm.

Neuronal survival analyses and immunofluorescence staining quantifications were performed in the ventrolateral and ventromedial region of the ventral horn in the spinal cord, where the motor neurons are located, as demonstrated in Figure 1.

#### 2.4.1. Toluidine blue staining to assess neuronal survival

For motoneuron counting, slides were kept for 30 min at room temperature and then stained with 0.05% toluidine blue for 30 s, dehydrated, diaphonized, and mounted with Entellan (Merck) and coverslip.

Motoneurons located in the ventrolateral and ventromedial nuclei of the spinal cord ventral horn were counted on alternate slides (Supplementary Figure 1). Counting was performed on both sides (right and left) of the spinal cord, and only cells with visible nuclei were counted.

To correct double neuron counts, because the same cell can be present in two sections, the formula of Abercrombie was used (Abercrombie and Johnson, 1946):


N=n.t/(t+d)

N is the corrected number of counted neurons, n is the counted number of cells, t is the thickness of the sections (12 μm), and d is the average diameter of the cells. Due to the possibility of differences in cell size among experimental conditions, the value of d was calculated specifically for each experimental group.

For statistical analysis, the sum of the sides was considered, and the ratio between NTG mice and transgenic mice of the different experimental groups was calculated to express the motoneuron loss due to the progression of ALS.

#### 2.4.2. Immunofluorescence

Immunofluorescence was evaluated in three representative alternate sections of the lumbar spinal cord per specimen. After blocking with 3% BSA (bovine serum albumin) in 0.1 M PB for 45 min, slides were incubated with primary antibodies against GFAP, Iba1, synaptophysin, MHC-I, GAD65, VGLUT-1, and AMPA receptor (Table 3) diluted in an incubation solution (1.5% BSA and 0.2% Tween in 0.01 M PB) and incubated for 4 h at room temperature. After washing with 0.01 M PB, the secondary antibodies (Table 3) were applied and incubated for 45 min. Sections were then rinsed in 0.01 M PB and mounted in a mixture of glycerol/PB (3:1).

**TABLE 3:** Antibodies used for immunofluorescence.

Primary antibody	Host	Manufacturer	Dilution	Catalog number	Secondary antibody
GFAP	Rabbit	Abcam	1:1500	ab7779	Alexa 488
Iba-1	Rabbit	Wako	1:750	01919741	Alexa 488
MHC-I	Rat	Biorad	1:200	MCA2398	Cy3
Synaptophysin	Rabbit	Novus Biologicals	1:1,000	NBPO2-25170	Cy3
GAD65	Mouse	Abcam	1:750	AB26113	Alexa 488
V-GluT-1	Rabbit	Synaptic Systems	1:1,000	135303	Alexa 488
AMPAr	Rabbit	Abcam	1:250	ab31232	Alexa 488
GFAP	Mouse	Abcam	1:400	ab279290	Cy3
NeuN	Mouse	Millipore	1:500	MAB377	CY3/Alexa488
CGRP	Rabbit	Millipore	1:1000	C8198	Alexa488

For quantification, one image of each side (right and left) was acquired for each slide, totaling 3 per animal, using a Leica fluorescence microscope (DM 5500, Wetzlar, Germany) equipped with a digital camera (DFC 345 FX, Wetzlar, Germany) using specific filters according to the secondary antibodies. A quantitative evaluation of labeling was carried out using the integrated density of pixel measurements in a fixed area corresponding to the ventral horn (Figure 1), as described by Oliveira et al. (2004).

Quantification was performed with ImageJ software (version 1.33u, National Institutes of Health, USA). The integrated density of pixels was calculated for each image, and the mean value for each experimental animal was calculated.

### 2.5. Classification of microglial cells

The microglia morphology was determined in the spinal cord lamina IX using ImageJ software (NIH, USA). The microglia cells were classified into five types, according to previous works (Diz-Chaves et al., 2012; Lopez-Rodriguez et al., 2015). Type I–cells with 2 or fewer cellular processes and type II–cells with 3 to 5 short branches. These two types are considered non-reactive microglia. Type III–cells with a small cell body and more than 5 branches; type IV–cells with a large soma and thicker and retracted processes and type V–cells with amoeboid soma, numerous and small processes. The last three types are considered reactive microglia.

### 2.6. Flow cytometry

After perfusion with PBS, the lumbar intumescences of the spinal cords were freshly dissected and processed for flow cytometry. For that, tissue was dissociated, and cells were isolated by Percoll gradient density. Further, cells were incubated with Live/Dead Kit reagents (Thermo Fisher, USA; catalog number: L34968) to assess cell viability. To analyze the cell polarization, microglial cells were labeled with anti-CD45-FITC, anti-CD11b-APC-Cy7, anti-CD206-PE-Cy7, anti-CD68-APC, anti-TNF-α-PE-Cy5, and anti-IL-10-PE using the True-Nuclear Transcription Buffer kit set according to the manufacturer’s instructions (Biolegend, USA; catalog number: 424401). Afterward, cells were washed and fixed, and 50,000 events were acquired in a NovoCyte Flow Cytometer (ACEA Biosciences, San Diego, CA, USA).

Data analysis was performed using the NovoExpress software (NovoExpress 1.3.0 software, ACEA Biosciences, San Diego, CA, USA).

To analyze the Cell phenotype, a tube without a label was made previously and used to position the quadrants in dot plots graphics. For this, we performed a blank tube for each Cell marker and determined the position of M1 and M2 microglia quadrants. After that, we performed the same analysis in the, respectively, labeled tubes. Cells were gated by a hierarchical gating strategy: single cells were gated first, followed by live cells; lastly, microglia were identified by their relatively low expression of CD45 (CD45^low^) and high CD11b (CD11b^+^). Subsequently, microglial cells which were TNFα^+^ and CD68^+^ were considered polarized toward a pro-inflammatory, M1 profile, and IL-10^+^ and CD206^+^ microglial cells were considered polarized to an anti-inflammatory, M2 profile.

### 2.7. RT–qPCR

After PBS perfusion, lumbar intumescences were dissected out immediately frozen in liquid nitrogen, and stored at −80 °C.

Total RNA was extracted using the QIAzol Lysis Reagent (Qiagen—cat. no 73,306), and reverse transcription was synthesized with 2.0 μg total RNA with the High-Capacity cDNA Reverse Transcription Kit (Applied Biosystems—Code: 4368814) according to the manufacturer’s instructions.

Following cDNA synthesis, real-time PCR was performed using a TaqMan Assay (Life Technologies) to evaluate the relative gene expression levels of the genes listed in Table 4.

**TABLE 4:** TaqMan assays used in the RT–qPCR analysis.

Gene	Assay ID
*β2m*	Mm00437762_m1
*Il1β*	Mm00434228_m1
*Il4*	Mm00445259_m1
*Ifnγ*	Mm01168134_m1
*Tnf*	Mm00443258_m1
*Nos2*	Mm00440502_m1
*Tgfβ*	Mm01178820_m1
*Arg1*	Mm00475988_m1
*Cgrp*	Mm00801463_g1
*Hprt*	Mm01545399_m1

For the PCR template, cDNA specimens in triplicate were used with TaqMan Gene Expression Master Mix (2 × ) (Life Technologies—Code: 4369016) and TaqMan assays (primers + hydrolysis probes) for the genes listed in Table 2. Amplification cycles were set as follows: 95°C for 10 min, followed by 45 cycles of 95°C for 15 s and 60°C for 1 min.

The reference gene was carefully selected based on unchanged expression under several experimental conditions. The HPRT1 reference gene was labeled with a VIC fluorophore, and the target genes were labeled with a FAM fluorophore. The entire procedure for the quantitative PCR was performed on the instrumentation platform MX3005P (Agilent, Santa Clara, CA, USA), and the results were calculated with MxPro software (v4.10; 2007, Stratagene).

For statistical analysis, the mean values of the three measurements for each animal were used as individual data for the relative quantification of the genes of interest using the 2^–ΔΔ Ct^ method (Livak and Schmittgen, 2001).

### 2.8. Statistical analysis

The quantitative results were analyzed using the mean value of each experimental group, and respective standard error, submitted to the One-way analysis of variance (ANOVA) followed by Bonferroni’s post-test (for neuronal survival, immunofluorescence), or Tukey’s post-test (RT-qPCR and Flow Cytometry). Rotarod, neurological score, weight, and microglial morphological classification were analyzed by the Two-way ANOVA, followed by the Tukey’s post-test. For the *post hoc* analysis of the differences between the means of the different groups, the Bonferroni’s test was used. All analyses were performed with the GraphPad Prism 8.0.1 software, and the significance level was established at *p* < 0.05. All data are expressed as the mean ± standard error of the mean (SEM).

## 3. Results

### 3.1. Effects of different doses of IFN β treatment on disease progression in presymptomatic animals

The assessment of behavioral changes during the presymptomatic disease progression was performed by the Rotarod test, neurological score, and measurement of body weight.

The rotarod performance test was performed to assess motor coordination and balance and is the most indicated to detect neuromotor alterations in the ALS model. The neurologic score shows signs of the clinical stage of the disease, and body weight can evaluate signs of muscle atrophy.

As expected, when the assessment was performed in the presymptomatic period, there were no differences in the time spent on the rotarod in the different experimental groups or significant variations in body weight, indicating that muscle atrophy had not yet occurred during this period. The evaluation of the neurological score was also not significantly different between groups (Supplementary Figure 2).

### 3.2. IFN β treatment increases local MHC-I expression in a dose-dependent manner

**FIGURE 2:**
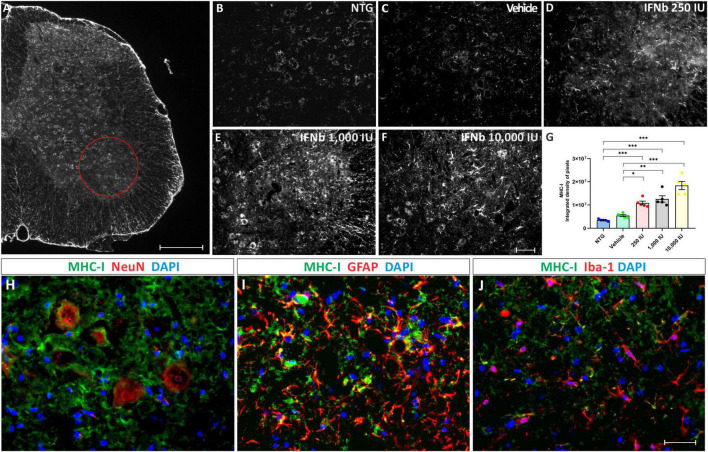
Anti-MHC-I immunostaining in the ventral horn of the spinal cord, in **(A)**, view of a spinal cord hemisection showing the pattern of anti-MHC-I immunostaining. The red circle indicates the Rexed lamina IX, where MHC-I expression was analyzed. Scale bar = 150 μm. Representative images of **(B)** NTG, **(C)** vehicle, **(D)** 250 IU, **(E)** 1,000 IU, and **(F)** 10,000 IU. **(G)** Quantification of the integrated density of pixels for MHC-I labeling. Figures illustrating double labeling of Dapi (blue) and MHC-I (green) along with **(H)** NeuN, **(I)** GFAP, and **(J)** Iba-1 (shown in red). Note that IFN β treatment is related to an increased MHC-I expression at all doses compared to the NTG and vehicle groups, mainly in motoneurons and astrocytes (One-Way ANOVA followed by Bonferroni’s post-test; *p < 0.05, **p < 0.01, and ***p < 0.001). Scale bar = 50 μm.

To assess whether IFN β treatment increases the expression of MHC-I, anti-MHC-I immunostaining was performed in the lumbar spinal cord of the experimental groups (Figure 2).

The disease progression, by itself, did not upregulate MHC-I immunostaining, as shown by the vehicle group. However, treatment IFN β increased the expression of MHC-I in the ventral horn of the spinal cord in a dose-dependent manner, compared to vehicle and NTG groups (NTG: 3510247 ± 183530; Vehicle: 5450908 ± 414581; 250 IU: 10858614 ± 800696; ***p < 0.001 compared to NTG and *p < 0.05 compared to Vehicle; 1,000 IU: 12589055 ± 1379600; ***p < 0.001 compared to NTG and **p < 0.01 compared to Vehicle; and 10,000 IU: 18376448 ± 1742132; integrated density of pixels, ***p < 0.001 compared to NTG and vehicle).

The results from the double labeling experiments combining MHC-I with neuronal and glial markers, revealed a higher degree of co-localization with motoneurons and astrocytes while showing lower expression in microglia.

### 3.3. IFN β treatment is neuroprotective to spinal motoneurons at the lowest and intermediate doses

The effects of the disease on neuronal survival, as well as the effect of treatment with IFN β were evaluated at 90 days of age, considered a transition phase between the asymptomatic and symptomatic period of the disease.

**FIGURE 3:**
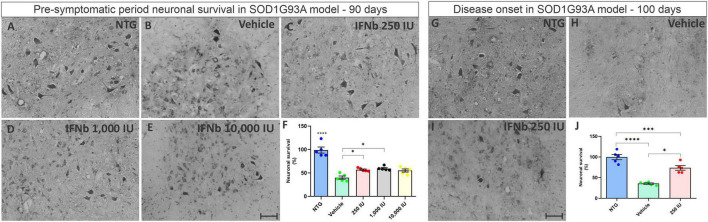
Cross-sections of the ventral horn of the lumbar spinal cord at the pre-symptomatic period. Representative images of the **(A)** NTG, **(B)** vehicle, **(C)** IFN β 250 IU, **(D)** IFN β 1,000 IU, **(E)** IFN β 10,000 IU, **(F)** graph showing the mean neuronal survival in the different experimental groups in the pre-symptomatic period (90 days of life). Representative images of the spinal motoneurons in the initial symptomatic period (100 days of life) in **(G)** NTG, **(H)** vehicle, **(I)** IFN β 250 IU experimental groups. **(J)** Graph showing the mean neuronal survival at 100 days. Even in the presymptomatic period, the animals with ALS showed a reduction in neuronal survival compared to the control group (****p < 0.0001). Importantly, IFN β treatment led to 31% up to 34% more preservation of motoneurons in 250 and 1,000 IU, respectively as compared to the vehicle group (*p < 0.05). In the initial symptomatic period, the vehicle group showed an even greater loss of motor neurons compared to the control group (****p < 0.0001), while the treatment with IFN β 250 IU was able to preserve about 40% more when compared to the vehicle (***p < 0.0005). Scale bar = 50 μm.

For analysis, the number of motoneurons present in the ventral horn of both sides of the lumbar spinal cord of each experimental animal was determined. For statistical analysis, the ratio (%) between TG animals and NTG animals was calculated (Figure 3). The results show that in the asymptomatic period, all TG mice displayed significant motoneuron loss when compared to the NTG counterpart (***p < 0.001). The mice treated with IFN β at the doses 250 and 1,000 IU showed ∼30% greater motoneuron survival when compared to the vehicle group (*p < 0.05), (NTG: 99.05 ± 6.514; Vehicle: 40.22 ± 3.406; 250 IU −57.16 ± 1.573; 1,000 IU −59.72 ± 1.945; 10,000 IU −55.38 ± 3.307). At disease onset (100 days of life), the vehicle group showed an even greater loss of motor neurons (****p < 0.0001), while the treatment with IFN β 250 IU was able to preserve about 40% more when compared to the vehicle group (***p < 0.0005) (NTG: 100.0 ± 6.461; Vehicle: 35.85 ± 1.7222; 250 IU −73.77 ± 5.573).

### 3.4. IFN β effects on synaptic changes during the progression of ALS

Synaptophysin is a protein localized in presynaptic vesicles and is used as a marker for synaptic coverage evaluation. The analysis of synaptic input changes resulting from the disease and the possible effect of treatment with IFN β was evaluated by immunostaining in the lumbar ventral horn, with the intensity of immunoreactivity represented by the integrated density of pixels.

**FIGURE 4:**
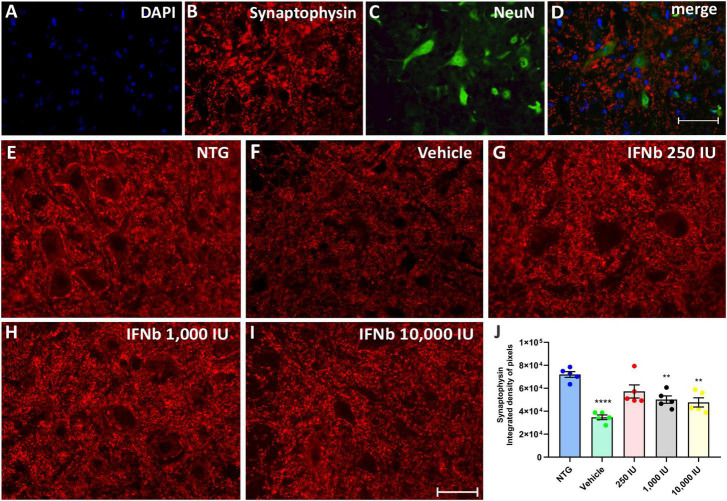
Anti-synaptophysin immunostaining in the ventral horn of the spinal cord. Double labeling counterstained with [**(A)**, blue] DAPI, showing [**(B)**, red] Synaptophysin, and [**(C)**, green] NeuN and **(D)** the merge, to evidence the immunostaining around the motoneurons located at the lamina IX of Rexed. Representative images of the **(E)** NTG, **(F)** vehicle, **(G)** 250 IU, **(H)** 1,000 IU, and **(I)** 10,000 IU. **(J)** Quantification of synaptophysin immunostaining in which the vehicle group showed a reduction of more than 50% of synaptophysin immunostaining when compared to the control group (NTG—non-transgenic counterpart) (****p < 0.0001). However, IFN β treatment at doses of 250, 1,000, and 10,000 IU preserved immunostaining by 31, 21, and 18%, respectively (250 IU: **p < 0.01; 1,000 IU: **p < 0.01 and 10,000 IU: **p < 0.01). Scale bar = 50 μm.

The quantification of the integrated density of pixels showed significant downregulation of labeling in the vehicle group (52%) when compared to NTG (NTG: 72041 ± 2526; Vehicle: 34732 ± 2024; ****p < 0,0001). The treatment at doses of 250, 1,000, and 10,000 IU led to synaptic preservation 31, 21, and 18% higher than the vehicle group (Figure 4), indicating that IFN β treatment, at all doses, is effective in synaptic preservation (250 IU: 57262 ± 5725; **p < 0.01; 50168 ± 3230; 1,000 IU: **p < 0.01 and 10,000 IU: 47731 ± 4011; **p < 0.01, respectively).

**FIGURE 5:**
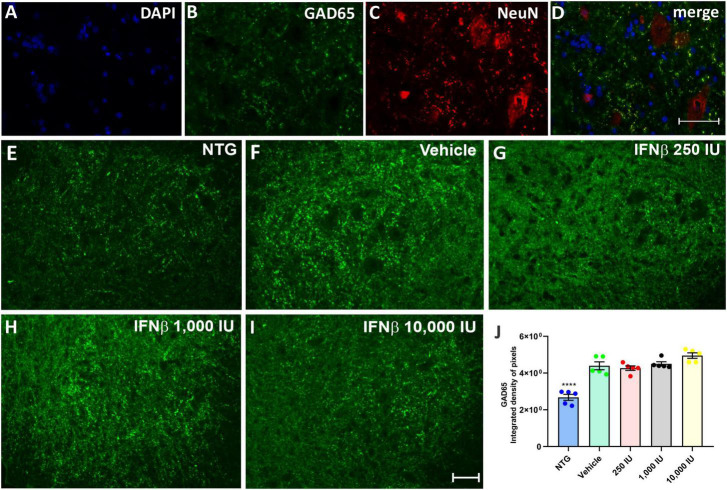
GAD65 immunostaining in the ventral horn of the spinal cord. Double labeling counterstained with [**(A)**, blue] DAPI, showing [**(B)**, green] GAD65, and [**(C)**, red] NeuN and **(D)** the merge, to evidence the immunostaining around the motoneurons located at the lamina IX of Rexed. Representative images of the **(E)** NTG, **(F)** vehicle, **(G)** 250 IU, **(H)** 1,000 IU, and **(I)** 10,000 IU. **(J)** Quantification of the integrated density of pixels for anti-GAD65 labeling, a marker of inhibitory inputs. The transgenic animals showed an increase in immunostaining as compared to the non-transgenic counterpart (NTG group; ****p < 0.0001). IFN β treatment in all studied doses did not show significant differences as compared to the vehicle. Scale bar = 50 μm.

The presence of inhibitory (GABAergic) and excitatory (glutamatergic) inputs were evaluated by immunolabeling with anti-GAD65 and anti-VGLUT-1 antibodies, respectively. The analysis by the integrated density of pixels showed that the animals in the vehicle group, as well as the treated animals, showed an increase of approximately 60% of GABAergic immunolabeling inputs, while there was no significant difference between the vehicle group and those treated with IFN β (Figure 5) (NTG: 2680 ± 1638; Vehicle: 4398 ± 2130; ****p < 0,0001; 250 UI: 4269 ± 1224; ****p < 0.0001; 1,000 UI: 4502 ± 1151; ****p < 0.0001 and 10,000 UI: 4953 ± 1494; ****p < 0.0001).

**FIGURE 6:**
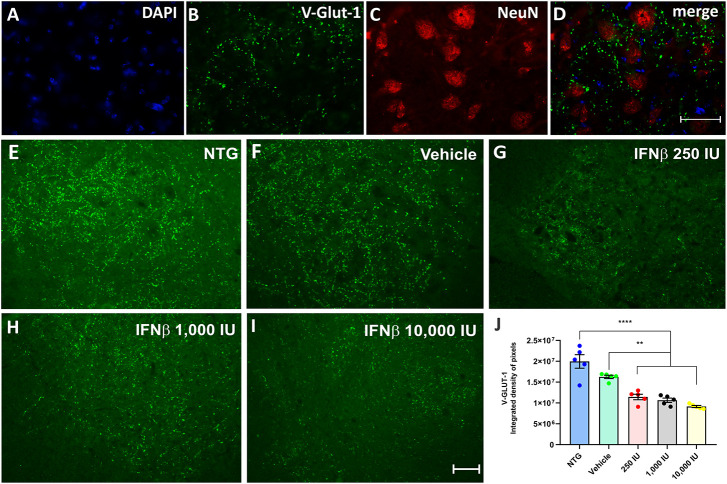
V-GLUT-1 immunostaining in the ventral horn of the spinal cord. Double labeling counterstained with [**(A)**, blue] DAPI, showing [**(B)**, green] V-GLUT-1, and [**(C)**, red] NeuN and **(D)** the merge, to evidence the immunostaining around the motoneurons located at the lamina IX of Rexed. Representative images of the **(E)** NTG, **(F)** vehicle, **(G)** 250 IU, **(H)** 1,000 IU, and **(I)** 10,000 IU. **(J)** Quantification of the integrated density of pixels for the V-GLUT-1 antibody, labeling excitatory inputs. The transgenic group showed a decrease in immunostaining as compared to the non-transgenic counterpart. The treatments further decreased immunostaining in a dose-dependent manner. (IFN β 250 UI: **p < 0.01; IFN β 1,000 UI: **p < 0.01, and IFN β 10,000 UI: ****p < 0.0001). Scale bar = 50 μm.

Glutamatergic vesicles, on the other hand, decreased in the vehicle and treated groups in a dose-dependent manner. The vehicle group displayed 16.6% less intense labeling than NTG, while the groups treated at doses of 250, 1,000, and 10,000 IU had a downregulation of 29, 36, and 45%, respectively, in comparison to the vehicle (Figure 6) (NTG: 19964154 ± 1621398; Vehicle: 16261809 ± 399116; 250 IU: 11438574 ± 664235; **p < 0.01; 1,000 IU: 10654462 ± 512523; **p < 0.01 and 10,000 IU: 9160399 ± 1352860; ****p < 0.0001).

### 3.5. IFN β treatment reduces astrogliosis and enhances astrocyte AMPA receptor expression

**FIGURE 7:**
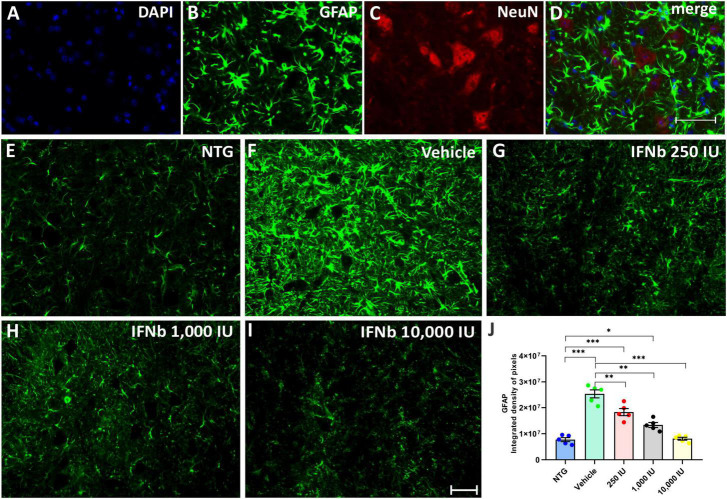
Anti-GFAP immunostaining in the ventral horn of the spinal cord. Double labeling counterstained with DAPI [**(A)**, blue], GFAP [**(B)**, green], and NeuN [**(C)**, red], and the merge **(D)** to evidence the immunostaining around the motoneurons located at the lamina IX of Rexed. Representative images of the **(E)** NTG, **(F)** vehicle, **(G)** 250 IU, **(H)** 1,000 IU, and **(I)** 10,000 IU. **(J)** Quantification of the integrated density of pixels for GFAP labeling, a marker for astroglial reactivity. Observe the disease-related GFAP upregulation in the Vehicle group, and its downregulation following the 250, 1,000 IU (**p < 0.01), and 10,000 IU IFN β treatment (***p < 0.001), compared to the vehicle group. The reactivity of the group treated with IFN β 1,000 IU nearly reached the level of the NTG (*p < 0.05), whereas the group treated with IFN β 10,000 IU practically equaled it. Scale bar = 50 μm.

Astrogliosis was analyzed by immunofluorescence using the antibody anti-GFAP (Figure 7). The quantification of the integrated density of pixels showed increased GFAP labeling in nearly all TG experimental groups when compared to NTG counterpart (Vehicle, and IFN β 250 IU: ***p < 0.001; IFN β 1,000 IU: *p < 0.05). However, decreased astrogliosis was depicted in all IFN β–treated groups in a dose-dependent manner compared to the Vehicle group (IFN β 250 IU, and IFN β 1,000: **p < 0.001; IFN β 10,000 IU: ***p < 0.001) (NTG: 7743741 ± 764713; Vehicle: 25311414 ± 1536500; 250 IU: 18313135 ± 1360993; 1,000 IU: 13372118 ± 944928; 10,000 IU: 8108219 ± 549412).

**FIGURE 8:**
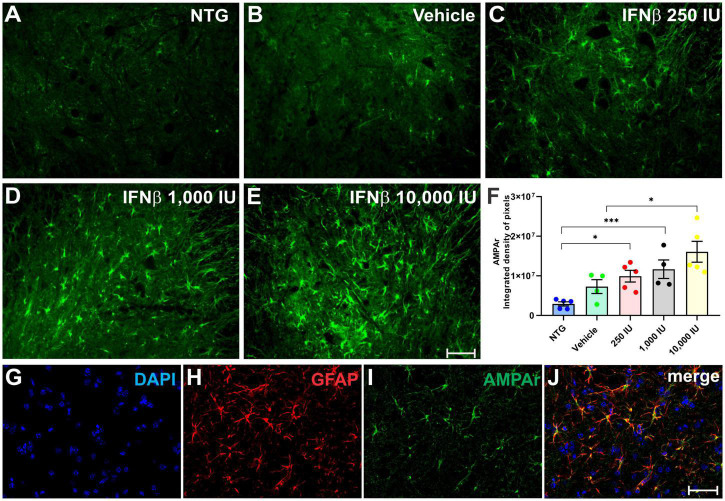
AMPAr immunostaining in the ventral horn of the spinal cord in **(A)** NTG, **(B)** vehicle, **(C)** IFN β–250 IU, **(D)** 1,000 IU, and **(E)** 10,000 IU. In **(F)**, Quantification of the integrated density of pixels for AMPAr labeling, depicting AMPA receptor presence in the gray matter. **(G)** Staining for cell nuclei (DAPI, blue), **(H)** immunolabeling for reactive astrocytes (GFAP, red), and **(I)** AMPAr (green), respectively. **(J)** Merge image evidencing that most expression of the AMPAr is present in astrocytes (NTG vs. IFN β 250 IU: *p < 0.05; NTG vs. IFN β 1,000 IU: ***p < 0.001; Vehicle vs. IFN β 10,000 IU: *p < 0.05). Scale bar = 50 μm.

AMPA receptor (AMPAr) expression was assessed in the spinal cord ventral horn (Figure 8), in which was detected an increased expression in the experimental groups treated with IFN β, particularly in thein 1,000 IU (**p < 0.01) and 10,000 IU (***p < 0.001) doses (NTG: 2917426 ± 520315; Vehicle: 7283183 ± 1757822; 2500 IU: 9914429 ± 1477247; 1,000 IU: 11674028 ± 2334274; 10,000 IU: 16071222 ± 2636748).

Double labeling of AMPAr and GFAP showed that most of the AMPAr immunolabeling was present in astrocytes.

### 3.6. IFNβ treatment changes microglia activation pattern

**FIGURE 9:**
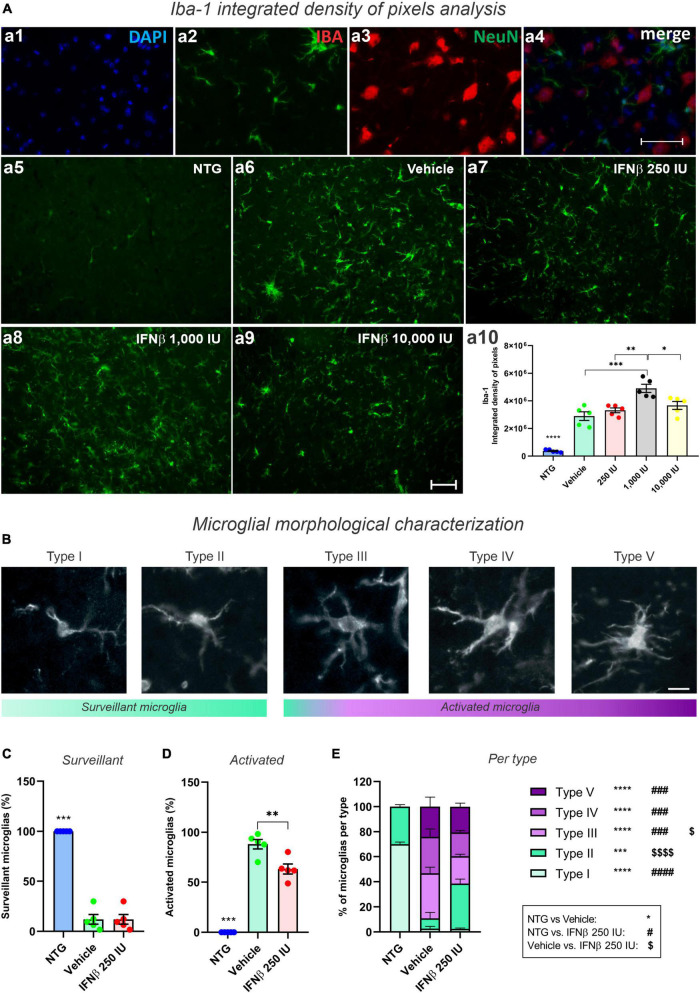
Morphological analysis of microglia. **(A)** Iba-1 integrated density of pixels analysis showing double labeling counterstained with (a1) DAPI, showing (a2) Iba-1, (a3) NeuN, and (a4) the merge to evidence the immunostaining around the motoneurons located at the lamina IX of Rexed. Representative photomicrographs of (a5) NTG, (a6) Vehicle, (a7) 250 IU, (a8) 1,000 IU, and (a9) 10,000 I. In (a10), Quantification of the integrated density of pixels for Iba-1 labeling. Increased microglial reactivity is observed in all TG when compared with NTG (****p < 0.0001). IFN β–1,000 IU group displayed the most intense Iba-1 upregulation (***p < 0.001, compared to the vehicle; **p < 0.01 compared to 250 IU, and *p < 0.05 compared to 10,000 IU). Scale bar = 50 μm. **(B)** Iba-1 positive microglia classified according to morphological aspects into surveillant (Types I and II) and activated (Types III, IV, and V). Scale bar = 50 μm. **(C)** Quantification of microglial cells into surveillant, and **(D)** activated. Note that IFN β treatment decreased the number of activated microglia when compared to the Vehicle group (**p < 0.001). **(E)** Detailed quantification of microglial types for each experimental group (NTG vs. vehicle: ***p < 0.001, ****p < 0.0001; NTG vs. IFN β 250 IU: ^###^*p* < 0.001, ^####^*p* < 0.0001; Vehicle vs. IFN β 250 IU: ^$^*p* < 0.05; ^$$$$^*p* < 0.0001). Two-way ANOVA, followed by Tukey’s post-test (*NTG vs. Vehicle; ^#^NTG vs. IFN β 250 IU; ^$^Vehicle vs. IFN β 250 IU).

Disease-related changes in microglial reactivity and the putative effects of IFN β were analyzed by Iba-1 immunostaining in the ventral horn of the lumbar spinal cord (Figures 9A, B). Transgenic mice showed increased anti-Iba-1 immunostaining as compared to the NTG group. IFN β–1,000 IU group, displayed the greater upregulation of Iba-1, surpassing the vehicle group (***p < 0.001) (NTG: 373292 ± 44782; Vehicle: 2897074 ± 310603; 250 IU: 3317348 ± 171326; 1,000 IU: 4904229 ± 296253; and 10,000 IU: 3670722 ± 287483).

In addition to the microglial reactivity analysis through Iba-1 immunostaining, we analyzed the cellular morphology which varies according to the activation degree (Figures 9C, D). It was observed that treatment with IFNβ led to a reduction in the number of activated microglia when compared to the Vehicle group (Figure 9E). Iba1-positive cells were classified into five different types: types I and II, considered surveillant microglia, and types III, IV, and V, considered activated.

As expected, in the NTG group 100% of the Iba1-positive cells were characterized as surveillant. In the TG mice, there was an increase in activated microglia, corresponding to 88% of the Iba1-positive cells in the vehicle group, and 63% in the IFN β−250 IU. Thus, IFN β treatment decreased the number of activated microglia in the time course of ALS (**p < 0.01).

Among the different morphologies, IFN β treatment increased the number of Type II, surveillant, microglia, and decreased Type III, activated counterparts. Overall, this data indicates that the IFN β at 250 IU shifts the morphological pattern of the microglia toward a less-activated profile.

Whereas the pro-inflammatory microglia reached 16% of the analyzed cells in the NTG group, such profile decreased to 6 and 3% in the vehicle and IFN β 250 IU, respectively (****p < 0.0001).

**FIGURE 10:**
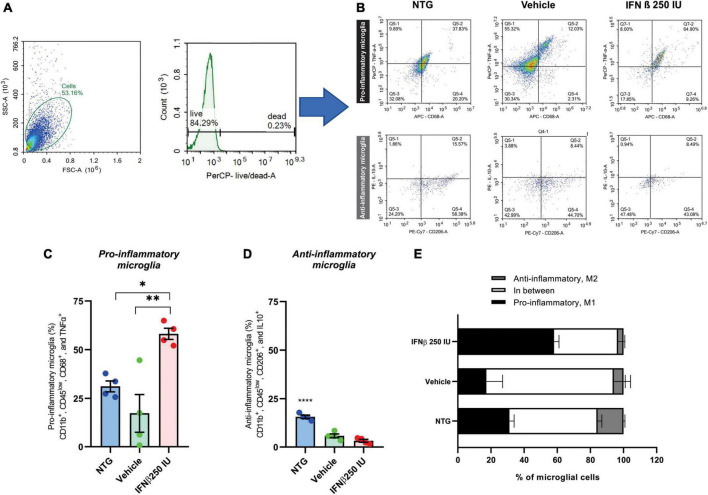
Effects of ALS and a low dose of IFN β administration on microglial phenotyping. **(A)** Scatter plot depicting size (*X*-axis, forward scatter, FSC-A) vs. cell granularity (*Y*-axis, side scatter, SSC-A) that allows the delimitation of the live microglial cells gate (herein corresponding to 84.29% of the total events acquired by the cytometer). **(B)** Representative flow cytometry dot plots showing TNFα and CD68 levels on pro-inflammatory microglia, and IL10 and CD206 on anti-inflammatory microglia from all experimental groups. Graphs with the percentages of **(C)** pro-, and **(D)** anti-inflammatory microglia. Note the increase in pro-inflammatory polarized microglia after IFN β administration (*p < 0.05, compared to NTG, and **p < 0.01 compared to vehicle). **(E)** Stacked bar graph summing up the percentages of polarized (anti- or pro-inflammatory microglia) and cells grouped in between these two polar phenotypes.

For a better understanding of the immunomodulatory effect of the IFN β treatment in ALS microglia, we proceeded to characterize the cytokine expression profile of such cells, using the flow cytometry technique (Figure 10).

Microglia activation has been associated with two dichotomic phenotypes, named M1 and M2. M1 microglia express pro-inflammatory mediators such as TNFα and displays specific surface markers such as CD68. M2 microglia is related to the expression of anti-inflammatory cytokines like IL-10, and display specific surface markers, such as CD206. Therefore, we quantified amongst live CD11b^+^ and CD45^low^ cells the percentage of cells that were polarized to the pro- (CD11b^+^, CD45^low^, CD68^+^, and TNFα^+^) and anti-inflammatory (CD11b^+^, CD45^low^, CD206^+^, and IL10^+^) profiles. In the NTG group, 31% of the cells were polarized toward the pro-inflammatory profile. Such polarization was reduced to ∼17% in the context of ALS but increased when IFN β (a pro-inflammatory cytokine) was administered, totaling ∼60% of the cells polarized do M1 profile (*p < 0.05 compared to NTG, and **p < 0.01 compared to vehicle) (NTG: 31.17 ± 2.810; Vehicle: 9.095 ± 3.592; IFN β 250 IU: 58.13 ± 2.876).

Of note, in NTG mice ∼50% of microglial cells were polarized either to pro- or anti-inflammatory profiles so that half of the microglial population analyzed displayed M1–M2 intermediate phenotypes. In the course of ALS, the percentage of polarized microglial cells was reduced, with ∼23% of cells either M1 or M2 polarized, resulting in ∼77% of the cells grouped in the continuum of intermediate phenotypes. The IFN β administration changed such a scenario back to NTG levels, leading to 60% of cells polarized to M1 or M2, favoring the M1 phenotype (*p < 0.05 compared to NTG, and **p < 0.01 compared to vehicle) (NTG: 1.995 ± 0.2347; Vehicle: 2.975 ± 1.709; IFN β 250 IU: 21.75 ± 5.837).

### 3.7. Analysis of gene expression in the early onset of ALS by RT–qPCR

**FIGURE 11:**
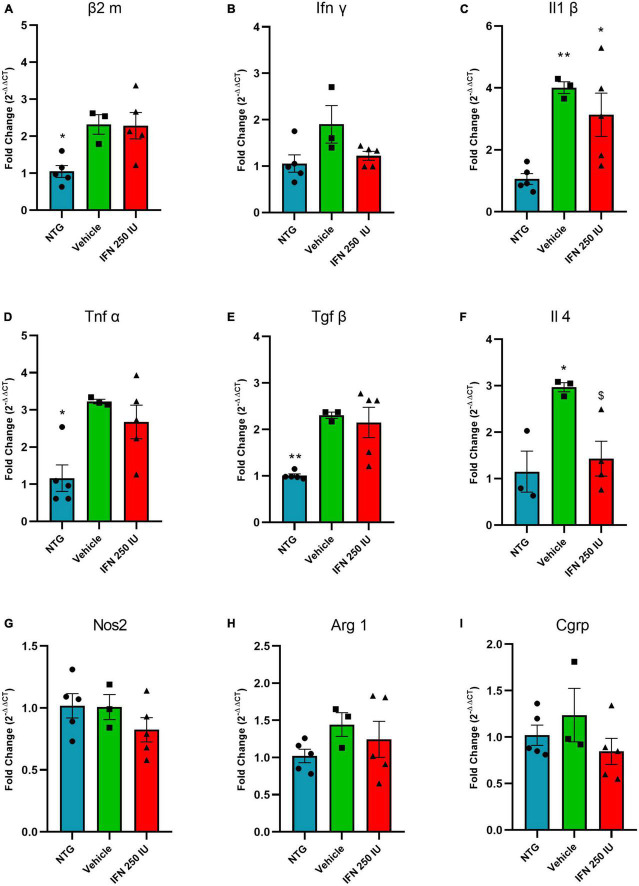
Relative quantification of gene expression of the β2 m **(A)**, Ifn γ **(B)**, Il1 β **(C)**, Tnf α **(D)**, Tgf β **(E)**, Il4 **(F)**, Nos2 **(G)**, Arg 1 **(H)**, and Cgrp **(I)** in the lumbar spinal cord of non-transgenic (NTG) and transgenic animals in the presymptomatic stage of ALS (*p < 0.05 and **p < 0.01 as compared to NTG; ^$^*p* < 0.05 compared to the vehicle).

Figure 11A shows the relative quantification of gene transcripts for the β2 microglobulin molecule, a subunit of MHC-I, present in the spinal cord of transgenic animals treated with 250 IU of IFN β or vehicle. The transgenic animals showed increased expression of β2 microglobulin compared to the NTG group; however, no significant difference was observed in comparison with the IFN β counterpart or the vehicle (NTG: 1.05 ± 0.17, Vehicle: 2.32 ± 0.26, IFN β 250 IU: 2.28 ± 0.35).

Figures 11B–D shows the relative quantification of gene transcripts for the pro-inflammatory cytokines INFγ, IL1β, and TNFα. In general, TG animals show increased levels of proinflammatory cytokines, which is considered an expected result due to the inflammation caused by ALS. IFN β treatment reduced IL-1β mRNA transcript levels (INFγ—NTG: 1.05 ± 0.18, Vehicle: 1.90 ± 0.40, IFN β 250 IU: 1.22 ± 0.09; IL1β–NTG: 1.06 ± 0.17, Vehicle: 4.00 ± 0.18, IFN β 250 IU: 3.13 ± 0.69, *p* < 0.01; TNFα—NTG: 1.16 ± 0.35, Vehicle: 3.22 ± 0.06, IFN β 250 IU: 2.67 ± 0.45, *p* < 0.05).

In addition to proinflammatory cytokines, the expression of gene transcripts for the anti-inflammatory cytokines TGFβ and IL4 was also verified (Figures 11E, F). We observed that TG animals showed increased expression of these cytokines compared to NTG; however, treatment with IFN β 250 IU was able to significantly reduce the levels of gene transcripts for the cytokine IL4 in comparison to the vehicle group (TGFβ—NTG: 1.00 ± 0.03, Vehicle: 2.30 ± 0.06, IFN β 250 IU: 2.15 ± 0.32, *p* < 0.01; IL4—NTG: 1.15 ± 0.44, Vehicle: 2.97 ± 0.09, IFN β 250 IU: 1.43 ± 0.37, *p* < 0.05).

The cytokine iNOS (Figure 11G) is used as a marker of the M1 profile of macrophages, characterized by the production of proinflammatory cytokines, while Arginase 1 (Figure 11H) represents the M2 profile of macrophages, characterized by the production of anti-inflammatory cytokines (Davis et al., 2013). Our results demonstrate that there were no significant differences between the experimental groups for the markers described above (iNOS—NTG: 1.01 ± 0.09, Vehicle: 1.00 ± 0.10, IFN β 250 IU: 0.82 ± 0.09; Arg1—NTG: 1.02 ± 0.09, Vehicle: 1.44 ± 0.16, IFN β 250 IU: 1.21 ± 0.24).

CGRP is a peptide related to the calcitonin gene and is expressed by motoneurons. Studies show that its upregulation is related to the susceptibility of the motoneuron to degenerate during the course of ALS (Ringer et al., 2012). Our results indicate the absence of significant differences between the experimental groups (CGRP—NTG: 1.02 ± 0.10, Vehicle: 1.23 ± 0.28, IFN β 250 IU: 0.84 ± 0.14; Figure 11I).

## 4. Discussion

Amyotrophic lateral sclerosis is a disease of fast progression, extremely disabling, and difficult to diagnose in its early stages. When the first clinical signs appear, close to half of the motoneurons have already degenerated. There is no preemptive treatment, and the drugs available for use by patients are riluzole and edaravone, which act by decreasing the excitotoxicity caused by glutamate and oxidative stress, respectively, prolonging the life of patients by a few months. More recently, Relyvrio, a drug combination of sodium phenylbutyrate and taurursodiol has been approved by the FDA as a therapeutic option for ALS (Paganoni et al., 2020, 2021, 2022).

Although sex dimorphism affects the incidence and prevalence of ALS in male subjects, controversial results have been reported (Hayworth and Gonzalez-Lima, 2009; McCombe and Henderson, 2010; Kim et al., 2012; Mancuso et al., 2012; Zwiegers et al., 2014; Pfohl et al., 2015; Curtis et al., 2017; Chiarotto et al., 2019; Tang et al., 2019). In addition, the bulbar onset is more observed in females than in males, which is associated with a faster disease progression (McCombe and Henderson, 2010; Kiernan et al., 2011). Remarkably, T-cell activation, antigen presentation molecules, microglial activation, and interferon pathway are more intense in female subjects (Keren-Shaul et al., 2017; Zrzavy et al., 2017; Gal-Oz et al., 2019). Female-derived immune cells, particularly macrophages, display elevated baseline expression of inflammatory genes (Gal-Oz et al., 2019). Based on the above discussed, in the present study, we evaluated female SOD1^G93A^ mice, similar to previous authors (Linares et al., 2013; Sironi, 2017; Chiarotto et al., 2022) that investigated the ALS pathophysiology.

Although excitotoxicity is a hallmark of the disease, the mechanisms underlying the pathogenesis of ALS are obscure which hampers the development of efficient therapeutical approaches. Since motoneuron loss is closely related to glial activation, microglial and astroglial reactivity have been thoroughly investigated, raising the possibility that mechanisms from innate and adaptative immunity contribute to the disease progression.

Indeed, it has been shown that both in mice and humans, there is a decrease in MHC-I expression by motoneurons, which, in turn, become more susceptible to degeneration (Staats et al., 2013; Song et al., 2016). Correspondingly, MHC-I upregulation by motoneurons has been described in a mouse model of ALS with slow disease progression (SOD1^G93A^ in C57Bl/6J background). Contrarily, a low MHC-I presenting mouse strain (SOD1^G93A^ in 129Sv background) displays fast disease progression (Nardo et al., 2013).

Herein, we administered IFN β in SOD1^G93A^ transgenic mice to increase MHC-I expression during the presymptomatic phase of ALS. Using immunofluorescence, we evaluated the expression of MHC-I in the lumbar segment of the spinal cord of NTG and TG mice treated with different doses of IFN β. Our results show that IFN β upregulates MHC-I expression in a dose-dependent manner. The immunostaining for MHC-I was significantly higher in the treated groups compared to the vehicle and NTG equivalents, mainly in motoneurons and astrocytes. Such data corroborate with the previous results of Zanon et al. (2010), which described the effectiveness of IFN β in significantly increasing the expression of MHC-I and the positive correlation of such upregulation and the regenerative success in response to axonal injury (Fuchs, 2012). On the other hand, the analysis of the β2 microglobulin gene transcripts showed that there is an upregulation in the β2m mRNA due to the disease; however, there was no significant increase in β2m mRNA after treatment with IFN β. But, in turn, IFN β treatment increased the expression of the MHC-I molecule in the gray matter of the ventral spinal cord, where motor neurons and surrounding glial cells are located. The fact that the entire lumbar spinal cord was processed for RNA extraction (including white matter) may explain the difference between immunofluorescence and RT-qPCR results. Furthermore, it is known that the mRNA-protein correlation is not always linear, and the possible discrepancy is also typically attributed to other levels of regulation between the transcript and the final protein product (Maier et al., 2009). We, therefore, suggest that the differential expression of MHC-I occurs locally in the lumbar spinal cord, specifically in the region where motor neurons are located, probably exercising its neuroprotective role there.

The dose-related increase in MHC-I expression is coupled with an increased motoneuron survival at the pre-symptomatic stage, which is maintained until the initial symptomatic stage at 100 days of life. The studies by Staats et al. (2013) with SOD1 transgenic mice and SOD1-knockout mice for β2-microglobulin (β2m), an obligatory subunit of the MHC-I molecule, reinforce that MHC-I is involved in slowing down the progression of ALS: the results show that SOD1-β2m knockout animals present faster disease progression.

Our finding demonstrates that IFN β, and consequently the increase in MHC-I expression, contributes to the preservation of motoneurons before the onset of the disease, implicating a better quality of life and possible improved lifespan.

Such neuroprotection exerted by MHC-I may be related to the downregulation of the natural killer (NK) T limphocyte cell activation. NK cells survey MHC-I expression, which in turn regulates cytotoxic response (Borrego et al., 2002). In healthy cells, including motoneurons, normal MHC-I expression protects against NK cell damage. Interestingly, ALS spinal cord motoneurons downregulate MHC-I expression, making them more vulnerable to NK-mediated cytotoxicity (Song et al., 2016; Figueroa-Romero et al., 2022). The relationship between MHC-I loss and NK cell damage in ALS is supported by the “missing self” hypothesis (Ljunggren and Kärre, 1990), which reports that NK cells recognize and destroy cells that show decreased MHC-I expression, which is a common feature of infected and transformed cells after viral infection (Borrego et al., 2002). Other studies have found that NK cell depletion slows motoneuron degeneration, delays motor impairment, and downregulates microglial activation and regulatory T cell infiltration (Garofalo et al., 2020; Appel et al., 2021). In line with the above discussed, other studies have demonstrated that the increase in MHC-I expression delays the progression of ALS symptoms and protects motoneurons from the toxic effects of astrocytes, suggesting that MHC-I expression also modulates the susceptibility to toxicity caused by reactive astrocytes (Nardo et al., 2016; Song et al., 2016).

We then analyzed astrogliosis by quantifying immunolabeling GFAP-positive astrocytes. The GFAP is a protein constitutively expressed by astrocytes and it is broadly used as a marker for astrogliosis (Vargas and Johnson, 2010). Through the quantification of the integrated density of pixels analysis, we observed that IFN β treatment decreased astrogliosis. In line with our data, the study by Song et al. (2016) demonstrated that ALS patients displayed a decrease in MHC-I levels in motoneurons and suggested that reactive astrocytes secrete stress inducers from the endoplasmic reticulum, which causes a decrease in MHC-I levels, thus making the neurons more susceptible to astrocyte-induced excitotoxicity.

Besides the signaling interplay that takes place between astrocytes and motoneurons during ALS, astrocytes also play a pivotal role in the CNS as regulators of neurotransmitter homeostasis, as they re-uptake synaptic-released neurotransmitters, such as glutamate. In ALS glutamate re-uptake by astrocytes is compromised (Boillée et al., 2006), and an exacerbated activation of the AMPA receptor (AMPAr) has been reported in diseased motoneurons (Antonella Gasbarri, 2014). Overall, such changes increase calcium permeability and influx to the cytoplasm contributing to motoneuron degeneration (Kwak et al., 2010; Kia et al., 2018; Sengupta et al., 2019; Gregory et al., 2020).

In the present work, the possible effect of IFN β in glutamate clearance was performed by analysis of the immunoreactivity against AMPAr in SOD1^G93A^ mice. The results indicate a significant increase in labeling in treated animals as compared to NTG equivalents, which is in line with previous literature (Kia et al., 2018; Sengupta et al., 2019; Gregory et al., 2020). The IFN β-treated groups showed a significantly higher density of AMPAr immunostaining in a dose-dependent manner. Complementarily, AMPAr upregulation was found to co-localize with GFAP-positive astrocytes.

In this regard, previous data suggest that astrocytes may be involved in the regulation of the neuronal expression of GluR2, a subunit of the AMPA receptor that mediates the vulnerability of neurons to calcium intake (Van Damme et al., 2007; Roopali Yadav et al., 2017). Importantly, the present results show that IFN β treatment decreased, in a dose-dependent manner, the astrocyte reactivity, while increasing the expression of AMPAr. Such correlation indicates a relationship between AMPAr expression and glial neuroprotective behavior.

The stabilization of reactive astrogliosis by IFN β administration is also related to the preservation of synaptic terminals in apposition to spinal motoneurons. The analysis of anti-synaptophysin immunostaining on the lamina IX of Rexed showed that the treatment at the three different doses contributed to the preservation of synaptic coverage of spinal motoneurons. Several studies have reported that SOD1^G93A^ animals, as well as ALS patients, suffer a reduction in synaptic coverage, mainly in spinal cord motoneurons, from the onset of symptoms (Ince et al., 1995; Ikemoto et al., 2002; Sunico et al., 2011), reaching up to 80% loss in end-stage disease (Chiarotto et al., 2019, 2022). It is suggested that this reduction in synaptic coverage is an initial step toward neurodegeneration, occurring simultaneously with the onset of the symptoms. In this sense, IFN β treatment and increased MHC-I expression contributed to the spinal synaptic circuitry preservation, which is in line with previous studies (Oliveira et al., 2004; Zanon and Oliveira, 2006; Zanon et al., 2010).

The fate of excitatory and inhibitory inputs was evaluated through immunostaining anti-VGLUT-1 and anti-GAD-65. The quantification of immunolabeling showed a decrease in glutamatergic synapses in the vehicle group in comparison to the NTG group suggesting a protective response to avoid the excitotoxicity exerted by glutamate. IFN β treatment further reduced glutamatergic immunolabeling by 45% in comparison to the vehicle counterpart. The same pattern of synaptic detachment has been described in axotomy models, where preferential retraction of excitatory synapses after the injury is observed, reflecting a synaptic reorganization, to favor axonal regeneration (Lindå et al., 2000; Spejo et al., 2019). This is in line with the hypothesis that the increased expression of AMPAr in astrocytes by MHC-I upregulation is related to decreased glutamate concentration in the motoneuron vicinity, thus reducing excitotoxicity, resulting in motoneuron survival. This hypothesis is reinforced by the study of Lalancette-Hebert et al. (2016), which reported that α-motoneurons receiving excitatory proprioceptive stimuli are more susceptible to ALS-induced degeneration. Contrarily, γ-motoneurons, which lack monosynaptic excitatory inputs from Ia afferent fibers, are disease resistant and spared.

Regarding GABAergic inputs (anti-GAD65 staining), we observed that TG animals showed greater immunoreactivity as compared to NTG animals, but treatment with IFN β did not significantly change that scenario, indicating that the plasticity of inhibitory inputs might be influenced by other mechanisms than only the MHC I upregulation.

Besides astrocytes, microglia have also been implicated in playing a role in ALS (Clarke and Patani, 2020). Microglia are highly motile cells constantly scanning their surroundings by extension and retraction of their processes (Nimmerjahn et al., 2005). These cells respond to a plethora of stimuli becoming activated and undergoing graded changes in their morphology, and gene expression (London et al., 2013; Chiarotto et al., 2019). Since neurodegeneration triggers neuroinflammation, microglial cells also undergo profound changes in the time course of ALS (Clarke and Patani, 2020). Using spatial transcriptomics, Maniatis et al. (2019) reported that changes in microglial gene expression occurred before any modifications in the motoneurons, indicating that the etiology of the disease might be triggered in non-neuronal cells.

Herein, we found an upregulation of Iba-1 staining in all TG mice with no significant differences when comparing vehicle and IFN β treated groups. Although there is Iba-1 upregulation in the process of microglial activation, it has become increasingly common to associate the analysis of Iba-1 labeling with other techniques that bring more details about the degree of microglia activation or the pro- or anti-inflammatory profile they assume. In this sense, we proceed to analyze the IFN β treatment influence on the microglial morphology and cytokine expression through analysis of microglial typing and flow cytometry, respectively.

Concerning microglial morphology analysis, we observed that the NTG group displayed predominantly surveillant cells, type I and II, comprising cells with few branches and long, thin processes, as expected (Ohgomori et al., 2016). The vehicle group, on the other hand, presented about 89% of cells characterized as reactive, which presents cell body hypertrophy, shorter processes, and, in its most activated degree (type V), assumes an amoeboid morphology.

The morphological changes in microglia activation, such as the shortening of branches and hypertrophy of the cell body, have already been reported in Alzheimer’s Disease, Multiple Sclerosis (Rasmussen et al., 2007; Plescher et al., 2018), and ALS (Ohgomori et al., 2016). And IFN β treatment showed to influence the microglial morphology by enhancing the number of Type II microglia while reducing the number of Type III, activated, microglia.

Coupled with microglial morphological changes, we analyzed the inflammatory profile of microglia through the flow cytometry technique. Activated microglia have been routinely classified into two opposing phenotypes, the M1, pro-inflammatory, and the M2, anti-inflammatory. However, microglia assume graded responses when activated, establishing a continuum of intermediate phenotypes between M1 and M2 (Brites and Vaz, 2014; Geloso et al., 2017; Clarke and Patani, 2020). In this regard, we comparatively quantified polarized M1 and M2 microglia, together with intermediately polarized cells. Across all experimental groups, we observed the expression of both M1 and M2 markers. Interestingly, in the NTG group, approximately half of the microglia displayed an undifferentiated phenotype, constituting nearly 80% of the total microglial population in the vehicle-treated group. Despite their morphological activation being predominantly classified as type IV and V, indicating an activated state, these microglia exhibited an ongoing dynamic alternation in their inflammatory phenotype. Such an increase in intermediately differentiated microglia is in line with previous reports (Geloso et al., 2017; Maniatis et al., 2019; Clarke and Patani, 2020), that even show that the profile changes during disease progression (Liao et al., 2012; Geloso et al., 2017; Maniatis et al., 2019; Clarke and Patani, 2020).

In the context of ALS, many neuroprotective strategies that focus on modulating microglia reactivity attempt to improve clinical and/or pathological features (Geloso et al., 2017; Clarke and Patani, 2020). In this context, the administration of IFN β in the presymptomatic stage of ALS, is related to an increase in pro-inflammatory microglia polarization, yielding ∼60% of M1 polarized microglia. Notably, alongside the polarization toward the M1 phenotype, we observed significant alterations in microglial morphology. These findings demonstrate that IFN β treatment induces the production of pro-inflammatory cytokines and, intriguingly, simultaneously exerts a modulatory effect on microglial morphology, leading to a less activated state. Notably, microglia exhibiting a moderately activated morphological state, particularly type III, maintain their capacity to produce pro-inflammatory cytokines (Gaudet and Fonken, 2018), which were the most notable after treatment in the RT-PCR analysis.

Being IFN β a pro-inflammatory cytokine, the M1 polarization is expected, but due to its complex mechanism of action, it can mediate protective functions on microglia, as extensively reported for multiple sclerosis, such as debris clearance, reduction of inflammatory cells passing through the blood-brain barrier, and also stimulate the production of growth factors, creating the possibility for increased neuronal survival and repair (Kieseier, 2011; Scheu et al., 2019), which can be considered favorable, especially in the early stages of ALS.

*In vitro* studies by Beers et al. (2006) revealed that microglia with mSOD1^G93A^ mutation produce and release more superoxide and nitrite, inducing neuronal death, in addition to resulting in further activation of microglia with neurotoxic characteristics. In contrast, Persson et al. (2005) observed that polysaccharide (LPS)-activated mouse microglial cell cultures express GLT-1 glutamate uptake proteins and contribute to the removal of excess glutamate from the neuronal environment. Another *in vitro* study demonstrated that after optic nerve injury, microglia activated by T helper cells through IFN-γ stimulation have an increased ability to remove neurotoxic extracellular glutamate (Shaked et al., 2005). Both studies, therefore, suggest that microglial activation, in certain contexts, can contribute to the reduction of excitotoxicity exerted by the excess of glutamate in the extracellular environment, acting in specific situations, with a neuroprotective role.

Moreover, when we analyzed the expression of the proinflammatory cytokines TNFα and IL1β, the TG animals showed overall upregulated gene expression of such cytokines, which were not upregulated by IFN β treatment, showing that punctual regulations of pro-inflammatory molecules around diseased motoneurons did not lead to overall neuroinflammation in the spinal cord, reducing putative negative effects of extended inflammation in the context of the disease (Henkel et al., 2009; Chiarotto et al., 2019; McCauley and Baloh, 2019).

Regarding the expression of anti-inflammatory cytokines, we observed an increase in the gene transcript levels of TGFβ and IL-4 in vehicle-treated animals. Such an increase has already been reported in other studies, indicating a direct relationship with the progression of ALS, where such cytokines can act by enhancing neurodegenerative inflammatory responses through microglia and T cells, contributing to the acceleration of disease progression (Endo et al., 2015; Chiarotto et al., 2019; Scheu et al., 2019; Polverino et al., 2020). In our analyses, the group treated with IFN β demonstrated reduced levels of gene transcripts for IL4 compared to the vehicle group. The reduction of IL4 has already been observed in other studies, which demonstrated, in multiple sclerosis models, that treatment with IFN β acts by reducing the expression of such anti-inflammatory cytokine (Schluep et al., 1999; Andersson et al., 2011; Kvarnström et al., 2013).

## 5. Conclusion

In conclusion, our results demonstrate that a low dosage of IFN β shows therapeutic potential in the presymptomatic phase of ALS, by increasing MHC-I expression, resulting in neuroprotection and immunomodulation. In addition, IFN β shifted microglia morphology and changed the molecular profile of activated microglia toward a pro-inflammatory pattern.

Influence of interferon β treatment also increased AMPAr expression in astrocytes, which could contribute to a decrease in neuronal toxicity, resulting in a higher motoneuron survival rate observed.

The RT–qPCR results indicated an increase in gene transcripts in TG mice of pro- and anti-inflammatory cytokines, with a significant influence of IFN β treatment on the reduction of IL4 expression, corroborating previous studies carried out in multiple sclerosis models. Further, treatment with IFN β preserved synaptic terminals, with a particular effect on excitatory, VGLUT-1-positive, terminals.

Overall, treatment with IFN β protects motoneurons from ALS-induced degeneration and acts on synaptic preservation, reducing astrogliosis. Further analyses are needed to address the persistence of the positive effects in the symptomatic phase of the disease, as well as to understand the mechanisms of action and possible combinations with other molecules that may increase ALS patients’ lifespan.

## Data availability statement

The raw data supporting the conclusions of this article will be made available by the authors, without undue reservation.

## Ethics statement

The animal study was approved by the Institutional Committee for Ethics in Animal Experimentation (CEUA, Institute of Biology, UNICAMP), protocol no (5323–1/2019). The study was conducted in accordance with the local legislation and institutional requirements.

## Author contributions

AT, LC, and GC conceived and performed experiments, analyzed data, and wrote the manuscript. AT and LO performed the flow cytometry experiments. AO conceived experiments, analyzed data, obtained funding, and wrote the manuscript. All authors contributed to the article and approved the submitted version.

## Footnotes

1
https://resources.jax.org/guides/guide-working-with-als-mice
