# Corrigendum: Neuroprotection by upregulation of the major histocompatibility complex class I (MHC I) in SOD1^G93A^ mice

**DOI:** 10.3389/fncel.2024.1493884

**Published:** 2024-09-23

**Authors:** Ana Laura M. R. Tomiyama, Luciana Politti Cartarozzi, Lilian de Oliveira Coser, Gabriela Bortolança Chiarotto, Alexandre L. R. Oliveira

**Affiliations:** Department of Structural and Functional Biology, Institute of Biology—University of Campinas (UNICAMP), Campinas, Brazil

**Keywords:** amyotrophic lateral sclerosis, IFN β, ALS therapy, MHC-I, gliosis, neuroprotection

In the published article, there was an error in Figure 6 as published. The figure was published without the letters that identify each image. The corrected Figure 6 and its caption appear below.

**Figure 6:**
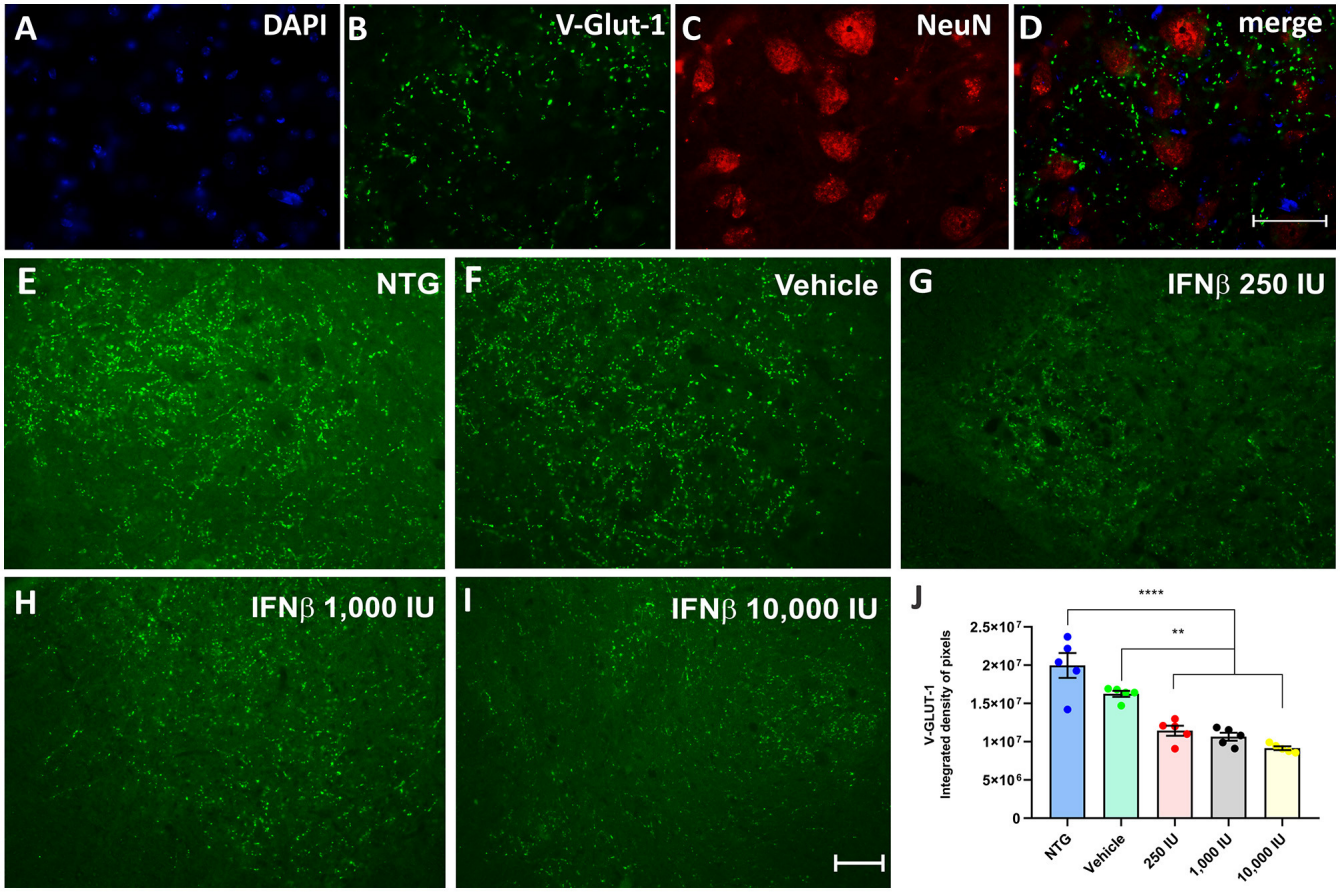
V-GLUT-1 immunostaining in the ventral horn of the spinal cord. Double labeling counterstained with [**(A)**, blue] DAPI, showing [**(B)**, green] V-GLUT-1, and [**(C)**, red] NeuN and **(D)** the merge, to evidence the immunostaining around the motoneurons located at the lamina IX of Rexed. Representative images of the **(E)** NTG, **(F)** vehicle, **(G)** 250 IU, **(H)** 1,000 IU, and **(I)** 10,000 IU. **(J)** Quantification of the integrated density of pixels for the V-GLUT-1 antibody, labeling excitatory inputs. The transgenic group showed a decrease in immunostaining as compared to the non-transgenic counterpart. The treatments further decreased immunostaining in a dose-dependent manner. (IFN β 250 UI: **p < 0.01; IFN β 1,000 UI: **p < 0.01, and IFN β 10,000 UI: ****p < 0.0001). Scale bar = 50 μm.

The authors apologize for this error and state that this does not change the scientific conclusions of the article in any way. The original article has been updated.

