# Supplementary material for: Neuroprotection by upregulation of the major histocompatibility complex class I (MHC I) in SOD1G93A mice
Source: Front Cell Neurosci. 2023 Aug 30;17:1211486. doi: 10.3389/fncel.2023.1211486 (PMC10498468; doi:10.3389/fncel.2023.1211486)
Supplement: Supplementary file 1 [file Data_Sheet_1.docx]

**Supplementary material**


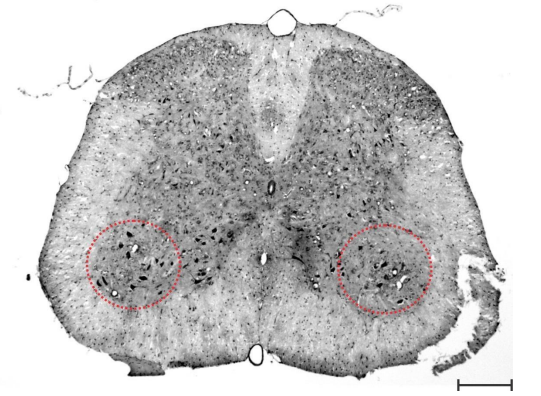


**Suppl. Figure 1-** Panoramic view of a nontransgenic mouse spinal cord, in cross-section, showing the location of the motoneurons that contribute to the sciatic nerve (red circles). The same region was used for the immunostaining quantification. Scale bar = 200 µm.


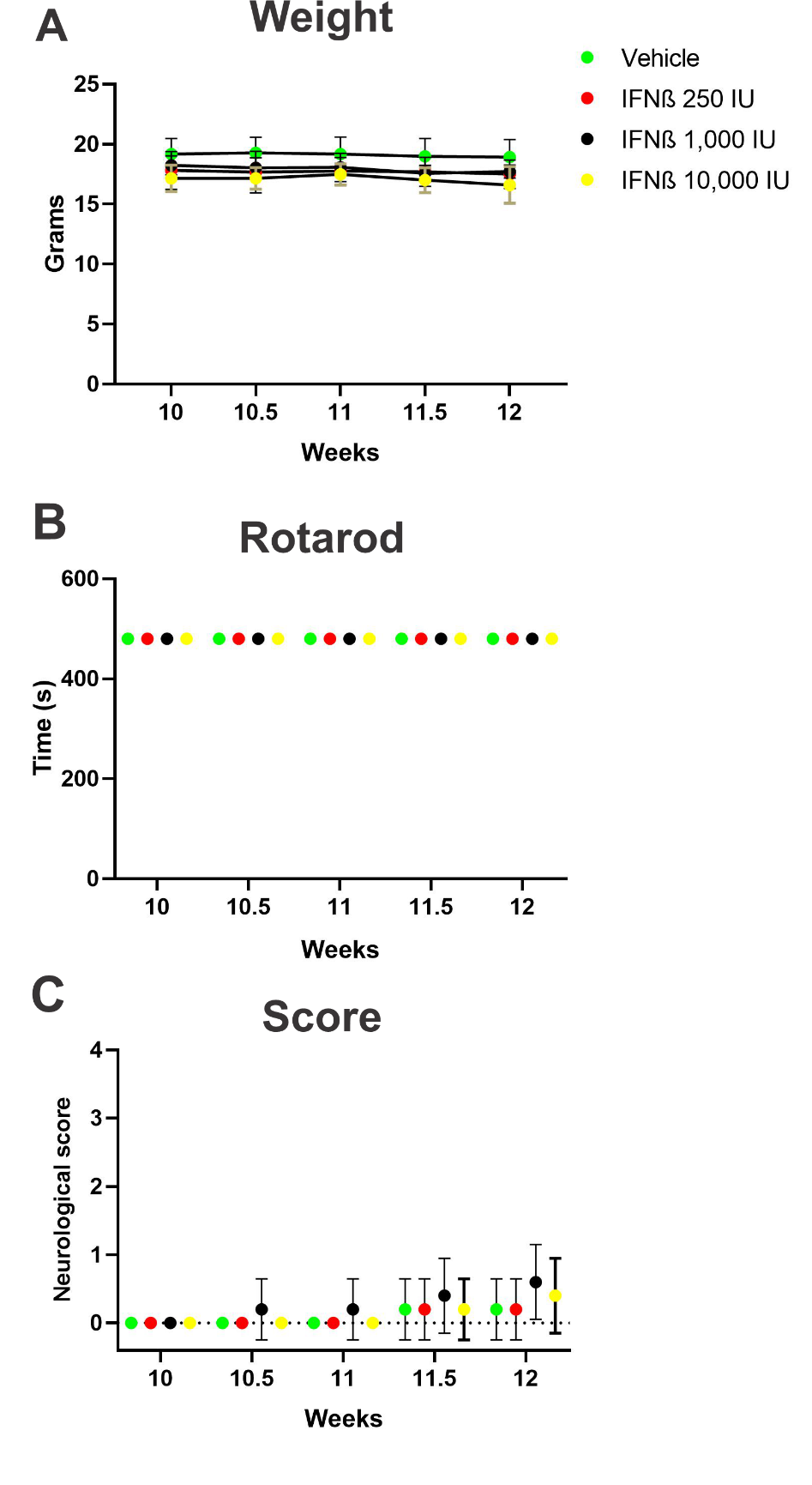


**Suppl. Figure 2-** Body weight (A), Rota-rod performance (B), and neurological score (C). No significant differences were detected in the different analyses between the experimental groups. Interferon b treatment: 250IU, 1,000IU, 10,000IU
